# Surgical Anatomy

**Published:** 1840-06

**Authors:** 


### SURGICAL ANATOMY AND OPERATIVE SURGERY.

Professor Gross is engaged in preparing a work on these subjects, which will comprise the anatomy of those parts of the body which are the chief seats of surgical operations, with an account of the most approved methods of performing them; to be illustrated with colored engravings and numerous wood cuts. It will be comprised in one volume, octavo, of about six hundred pages, and put to press early next spring.

We are happy to know that the Elements of Pathological Anatomy of this gentleman, meet with the approval of the profession wherever the work is disseminated.

We shall give a short analytical notice of it in an early number of the Journal. D.

